# Sex and Age Differences in Outcomes After Endovascular Thrombectomy of Patients With Acute Ischemic Stroke and Atrial Fibrillation: A Danish Nationwide Study

**DOI:** 10.1161/JAHA.125.048384

**Published:** 2026-08-07

**Authors:** Muath Alobaida, Deirdre A. Lane, Jakob N. Hedegaard, Fiona J. Rowe, Gregory Y. H. Lip, Thomas Truelsen, Boris Modrau, Claus Z. Simonsen, David Gaist, Stephanie L. Harrison, Søren P. Johnsen

**Affiliations:** ^1^ Liverpool Centre for Cardiovascular Science University of Liverpool, Liverpool John Moores University and Liverpool Heart & Chest Hospital Liverpool United Kingdom; ^2^ Department of Cardiovascular and Metabolic Medicine Institute of Life Course and Medical Sciences, University of Liverpool Liverpool United Kingdom; ^3^ Department of Basic Science, Prince Sultan Bin Abdulaziz College for Emergency Medical Services King Saud University Riyadh Saudi Arabia; ^4^ Department of Clinical Medicine Danish Center for Health Services, Aalborg University Aalborg Denmark; ^5^ Institute of Population Health University of Liverpool Liverpool United Kingdom; ^6^ Institute of Life Course and Medical Sciences University of Liverpool Liverpool United Kingdom; ^7^ Department of Cardiology, Lipidology and Internal Medicine with Intensive Coronary Care Unit Medical University of Bialystok Poland; ^8^ Department of Neurology University Hospital of Copenhagen, Rigshospitalet Copenhagen Denmark; ^9^ Department of Clinical Medicine University of Copenhagen Copenhagen Denmark; ^10^ Department of Neurology Aalborg University Hospital Aalborg Denmark; ^11^ Department of Clinical Medicine Aalborg University Aalborg Denmark; ^12^ Department of Neurology Aarhus University Hospital Aarhus N Denmark; ^13^ Department of Clinical Medicine Aarhus University Aarhus Denmark; ^14^ Research Unit for Neurology, Department of Neurology Odense University Hospital Odense Denmark; ^15^ Registry of Senior Australians (ROSA) Research Centre Caring Futures Institute, Flinders University Bedford Park Australia; ^16^ ROSA Research Centre South Australian Health and Medical Research Institute Adelaide Australia

**Keywords:** atrial fibrillation, endovascular thrombectomy, ischemic stroke, outcomes, reperfusion, sex differences, stroke, Ischemic Stroke, Atrial Fibrillation, Quality and Outcomes, Women

## Abstract

**Background:**

Sex disparities in outcomes after endovascular thrombectomy (EVT) for large vessel occlusion stroke in patients with atrial fibrillation (AF) remain unclear. This study examined sex differences in outcomes following EVT according to AF status.

**Methods:**

Using the nationwide Danish Stroke Registry, adults with first‐time ischemic stroke involving anterior and posterior circulation large vessel occlusion treated with EVT from 2015 to 2020 were included. Outcomes included 90‐day good functional outcome (modified Rankin Scale score ≤2), successful reperfusion (modified Thrombolysis in Cerebral Infarction ≥2b), symptomatic intracranial hemorrhage, and mortality. Multivariable logistic regression assessed associations between AF and outcomes stratified by sex, including sex‐and‐age interactions.

**Results:**

Among 2542 EVT‐treated patients (median age 72.5 years; 43.2% women), 729 (28.7%) had AF. Women with AF had higher odds of good functional outcome than women without AF (adjusted OR [aOR], 1.48 [95% CI, 1.10–1.99]). Men with AF had similar odds of good functional outcome as men without AF (aOR, 1.17 [95% CI, 0.90–1.53]) but higher odds of successful reperfusion (aOR, 2.04 [95% CI, 1.37–3.02]). No differences by sex or AF status were observed for excellent functional outcomes, symptomatic intracranial hemorrhage, or mortality. Among men, older men with AF was associated with higher odds of successful reperfusion compared with older men without AF (*P*_interaction_<0.001).

**Conclusions:**

Among patients undergoing EVT for large vessel occlusion stroke in Denmark, women with AF had higher odds of good functional outcome and men with AF showed higher successful reperfusion rates with advancing age. Safety and mortality outcomes were similar across groups with AF. These findings suggest age‐and sex‐specific influences on EVT outcomes associated with AF.

## Graphical abstract

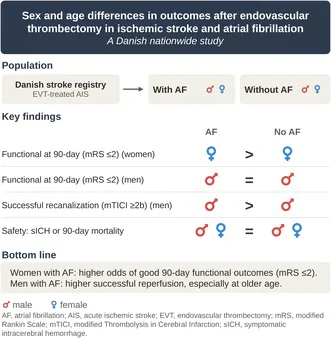

**Nonstandard Abbreviations and Acronyms**
CCI: Charlson Comorbidity Index
EVT: endovascular thrombectomy
mRS: modified Rankin Scale
NIHSS: National Institutes of Health Stroke Scale
PH2: parenchymal hemorrhage type 2
sICH: symptomatic intracranial hemorrhage

**Clinical Perspective**

**What Is New?**

- In a nationwide endovascular thrombectomy‐treated large‐vessel occlusion stroke cohort, women with atrial fibrillation had higher adjusted odds of good 90‐day functional outcome than women without atrial fibrillation, whereas no corresponding difference was observed in men.
- Among men, atrial fibrillation was associated with higher odds of successful reperfusion, particularly in older men, without differences in symptomatic intracranial hemorrhage or 90‐day mortality.

**What Are the Clinical Implications?**

- These findings suggest that atrial fibrillation identifies sex‐ and age‐specific patterns of response to endovascular thrombectomy, supporting tailored poststroke prognostication and recovery planning that integrates atrial fibrillation status with sex and age.

Atrial fibrillation (AF) is a leading comorbidity in patients with large vessel occlusion acute ischemic stroke.^1^ Irrespective of cause, endovascular thrombectomy (EVT) represents the gold‐standard treatment for large vessel occlusion‐related stroke.^2^ Patients with AF tend to be older, have more comorbidities,^3^ and often suffer from occlusions of large arteries and impaired collateral flow.^4^ Therefore, patients with AF have increased risk of serious disability and mortality after stroke.^5^, ^6^ In a pooled analysis of 6543 patients with acute ischemic stroke, patients with AF experienced worse outcomes at 90 days following EVT, despite a similar rate of successful reperfusion.^7^

There is growing interest from several studies (n=9) exploring sex disparities in clinical outcomes following EVT, which have yielded conflicting findings, showing worse or neutral outcomes for women compared with men.^8^, ^9^, ^10^, ^11^, ^12^, ^13^, ^14^, ^15^, ^16^ Women and older people are disproportionately affected by stroke and often experience worse outcomes.^11^, ^17^ Given that age is a risk factor for both stroke and AF, and that female patients with AF have a higher risk of stroke, understanding how sex and age influence EVT outcomes in patients with AF is crucial for identifying modifiable clinical and sociodemographic factors that may affect postacute functional recovery.^10^ This could inform personalized management strategies, including rehabilitation planning, secondary prevention measures, and targeted support based on age‐ and sex‐specific risks.

A systematic review and meta‐analysis of 993 603 patients demonstrated that female sex acts as a risk modifier, with women, particularly those aged >65 years, showing a significantly higher risk of stroke compared with men.^18^ These factors suggest that the relationship between AF status and EVT outcomes may not be uniform across age and sex strata, with older women especially vulnerable to poorer functional outcomes. However, to date none of the existing studies have specifically addressed the effect of AF on sex and age differences in outcomes following EVT in patients with AF compared with patients without AF. Therefore, this study investigated how sex and age affect the outcomes of EVT in patients with AF compared with without AF, in a nationwide cohort with complete follow‐up.

## METHODS

### Data Availability and Access

In line with Danish data protection regulation, data for this study cannot be shared publicly but may accessed via the Danish Health Data Authority and Statistics Denmark for researchers at authorized institutions. According to Danish law, neither ethical approval nor patient consent is required for registry‐based studies.

### Setting and Data Source

A nationwide population‐based observational study using the Danish Stroke Registry was conducted.^19^ Denmark has free access to health care for all residents through a tax‐funded health care system.^20^ The registry aims to capture all patients with acute stroke and transient ischemic attack aged ≥18 years admitted to a Danish hospital. Data are prospectively collected using standardized forms during admission, and registration is mandatory for all hospitals.^19^ The registry has a reported sensitivity and positive predictive value of the stroke diagnosis of 97% and 90%, respectively.^21^

### Study Population

The study population included all individuals (aged ≥18 years) admitted with a first‐time acute ischemic stroke recorded in the registry who underwent EVT or where EVT was attempted (unsuccessful) for large vessel occlusion between 2015 and 2020 in Denmark. Patients were excluded if they were ineligible for EVT during their admission, had a previous stroke diagnosis, immigrated to Denmark within the year preceding their stroke (as this limited the availability of prior health records), or had invalid information on residence or vital status (Figure 1).

**Figure 1:**
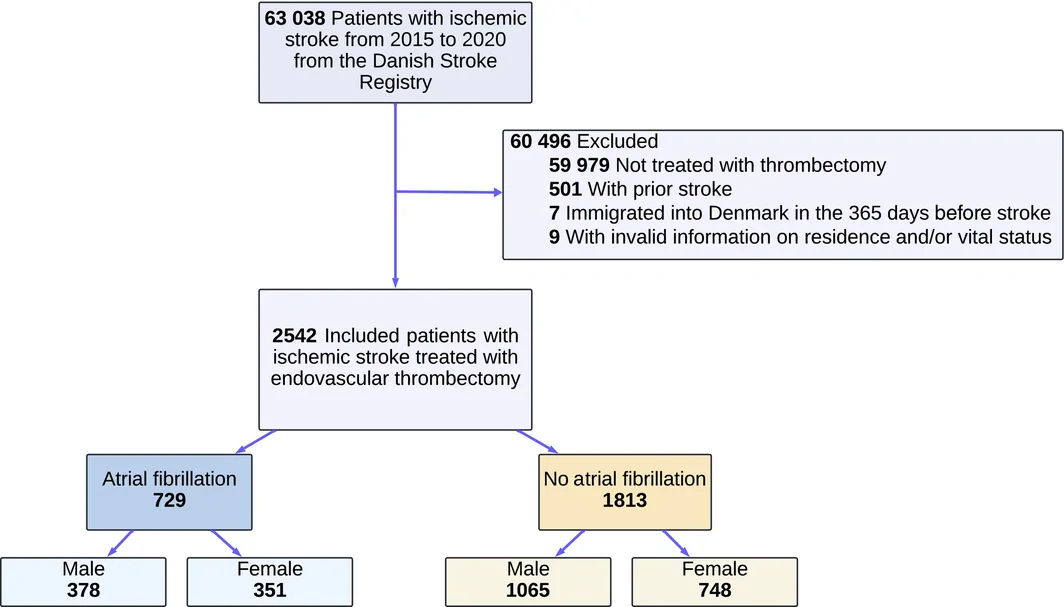
Flow diagram of selected patients with ischemic stroke who were treated with endovascular thrombectomy between 2015 and 2020, dichotomized by baseline AF status and sex. AF indicates atrial fibrillation.

### Outcomes

The primary outcome was good functional outcome (modified Rankin Scale [mRS] score ≤2) at 90 days post stroke. In Denmark, all patients treated with revascularization therapies are systematically followed up at 90 days either as outpatients or by structured telephone interview conducted by a neurologist or a stroke nurse trained and certified to score the mRS. Secondary outcomes included good functional outcome at discharge, excellent functional outcome defined as mRS score 0 to 1 at discharge and 90 days, successful reperfusion (modified Thrombolysis in Cerebral Infarction score of ≥2b), symptomatic intercerebral hemorrhage (sICH) within 90 days post stroke (defined as a parenchymal hemorrhage type 2 with National Institutes of Health Stroke Scale [NIHSS] scores increased ≥4 points or resulting in death) and all‐cause mortality within 90 days.

### Covariates

Stroke severity was measured using NIHSS at admission. Comorbidity burden was assessed with the Charlson Comorbidity index (CCI), categorized as low (0), moderate (1–2), or high (≥3). Civil status at time of admission was used to define cohabitation (cohabitant, lives alone, other/undisclosed). Immigration status distinguished immigrants and descendants of immigrants, based on national registry classification. This covariate was included because a Danish register‐based study has shown that immigrants experience lower rates of reperfusion therapy and longer delays to treatment,^22^ which may influence poststroke outcomes. Diagnosis of AF, diabetes, myocardial infarction, hypertension, previous stroke, and peripheral vascular disease were based on documented history of the condition or a diagnosis made during the current admission based on the *International Classification of Diseases, Tenth Revision* (*ICD‐10*) codes.^19^

### Statistical Analysis

Data were summarized into categorical variables using counts and percentages, and continuous variables were reported as medians (interquartile range). To assess the association between AF and no AF stratified by sex, multivariable logistic regression analyses were performed adjusting models for potential confounding factors: age, cohabitation, immigration status, admission NIHSS and CCI (0, 1–2, or ≥3).

Potential interaction by sex and age (with age dichotomized at the cohort median 72.5 years) was evaluated by stratum specific analyses on all included variables to allow for a balanced comparison across age groups and ensured statistical robustness while accounting for potential non‐linear relationships between age and outcomes. Additionally, *P* values for interaction between AF and sex were calculated by including the relevant interaction term in the regression models. Interaction terms with a *P* value of <0.05 were considered statistically significant. Missing data for covariates were reported in the respective table legends. Most variables had ≤5% missingness, thus no imputation was applied for these data.

All analyses were conducted using Stata (Release 16; StataCorp LLC.) The study followed the Strengthening the Reporting of Observational Studies in Epidemiology (STROBE) guideline.^23^

### Sensitivity Analysis

To assess the robustness of the observed association between AF and 90‐day good functional outcome (mRS ≤2) among women, sensitivity analyses were conducted. Specifically, logistic regression models were repeated with different covariate adjustments: (1) adjusting for age alone, and (2) adjusting for all selected covariates except age. This approach was used to explore the influence of age on the direction and magnitude of the association.

## RESULTS

There were 63 038 patients with acute ischemic stroke in the Danish Stroke registry from 2015 to 2020, of whom 2542 met the inclusion criteria for this study (Figure 1). There were 729 (28.7%) patients with AF and 1813 (71.3%) patients without AF. Patients with AF were significantly older (median age 77 [interquartile range 70–83.3] versus those without AF, 70 [58.7–77.3]), had more severe strokes (higher NIHSS score), greater comorbidity (higher CCI score), and a significantly higher prevalence of hypertension and myocardial infarction than patients without AF. Prevalence of diabetes and peripheral vascular disease were similar across groups. Patients with AF were more frequently treated with oral anticoagulation and were less frequently treated with intravenous thrombolysis compared with patients without AF (Table 1). Occlusion sites also differed between patients with and without AF; there was a significantly lower prevalence of internal carotid artery and basilar artery occlusions, but more middle cerebral artery‐M1 occlusions in patients with AF compared with patients without AF. Patients with AF had a significantly lower median (interquartile range) time from stroke onset to hospital arrival, groin puncture, successful reperfusion/end of procedure, and from arrival to successful reperfusion/end of procedure compared with patients without AF.

**Table 1:** Baseline Characteristics of the Danish Stroke Registry Cohort Stratified by Atrial Fibrillation Status

Characteristic	Atrial fibrillation (n=729)	No atrial fibrillation (n=1813)	*P* value
Male sex, n (%)	378 (51.8)	1065 (58.7)	0.001
Age group, y, n (%)			
<60	57 (7.8)	496 (27.4)	<0.001
60–69	123 (16.9)	412 (22.7)	
70–79	271 (37.2)	571 (31.5)	
≥80	278 (38.1)	334 (18.4)	
Admission National Institutes of Health Stroke Scale score, median (IQR)*	17 (12–22)	15 (9–20)	<0.001
Charlson Comorbidity Index, n (%)			
Low (0 points)	352 (48.3)	1146 (63.2)	<0.001
Moderate (1–2 points)	249 (34.2)	477 (26.3)	
High (≥3 points)	128 (17.6)	190 (10.5)	
Transient ischemic attack, n (%)	18 (2.5)	§§	§§
Hypertension, n (%)†	418 (58.1)	868 (48.6)	<0.001
Diabetes, n (%)‡	97 (13.4)	213 (11.9)	0.30
Myocardial infarction, n (%)§	81 (11.3)	124 (7.0)	<0.001
Peripheral vascular disease, n (%)‖	37 (5.3)	73 (4.1)	0.23
Occlusion site, n (%)			
Internal carotid artery	74 (10.2)	414 (22.8)	<0.001
Internal carotid artery—Tandem	80 (11.0)	186 (10.3)	0.59
Middle cerebral artery—M1	376 (51.6)	829 (45.7)	0.008
Middle cerebral artery—M2	174 (23.9)	382 (21.1)	0.12
Anterior cerebral artery—A1 or A2	20 (2.7)	43 (2.4)	0.59
Vertebral artery	§§	43 (2.4)	§§
Basilar artery	37 (5.1)	151 (8.3)	0.005
Posterior cerebral artery/other	38 (5.2)	86 (4.7)	0.62
Intravenous thrombolysis, n (%)	343 (47.1)	1108 (61.1)	<0.001
Anticoagulation therapy, n (%)	271 (37.2)	63 (3.5)	<0.001
Type of anesthesia, n (%)¶			
General anesthesia	522 (71.6)	1329 (73.3)	0.13
Conscious anesthesia	191 (26.2)	417 (23.0)	
Time metrics, h, median IQR			
Onset to hospital arrival#	3.3 (2.3–5.5)	3.7 (2.4–6.4)	0.003
Onset to groin puncture**	3.6 (2.6–5.9)	4.1 (2.8–6.7)	0.001
Onset to successful reperfusion/end of procedure††	4.6 (3.5–6.9)	5.3 (3.8–7.9)	<0.001
Arrival to successful reperfusion/end of procedure‡‡	2.8 (2.1–3.8)	3.2 (2.2–4.4)	<0.001

Education was defined according to the International Standard Classification of Education. Redemption of anticoagulation drugs within 6 months before stroke.

IQR indicates interquartile range.

* 102 missing.

^†^ 36 missing.

^‡^ 31 missing.

^§^ 38 missing.

^‖^ 75 missing.

^¶^ 83 missing.

^#^ 128 missing.

** 120 missing.

^††^ 120 missing.

^‡‡^ 124 missing.

^§§^ Number of cases are not reported in accordance with Statistics Denmark's data confidentiality policy.

### Sex‐Specific Baseline Characteristic Stratified by AF Status

Baseline and procedural characteristics of the cohort of patients receiving EVT stratified by AF and sex are shown in Tables S1 and S2 and Figure 1. Women were older than men in both groups with and without AF, with a higher proportion aged ≥80 years (Figure 2), had lower levels of education, and more often lived alone than their male counterparts. There was no difference in sex or AF status regarding CCI, median admission NIHSS score, thrombolysis administration, use of oral anticoagulation, or anesthesia type (Table S1). Women with and without AF had a higher prevalence of hypertension and lower prevalence of previous myocardial infarction compared with men in both groups with and without AF (Table S1). In patients without AF, women had a significantly lower rates of internal carotid artery, vertebral, and basilar occlusions but higher rates of middle cerebral artery‐M1 occlusions compared with men. In patients with AF, there was no significant sex differences in occlusion site, with middle cerebral artery‐M1 or M2 being the most common occlusion site.

**Figure 2:**
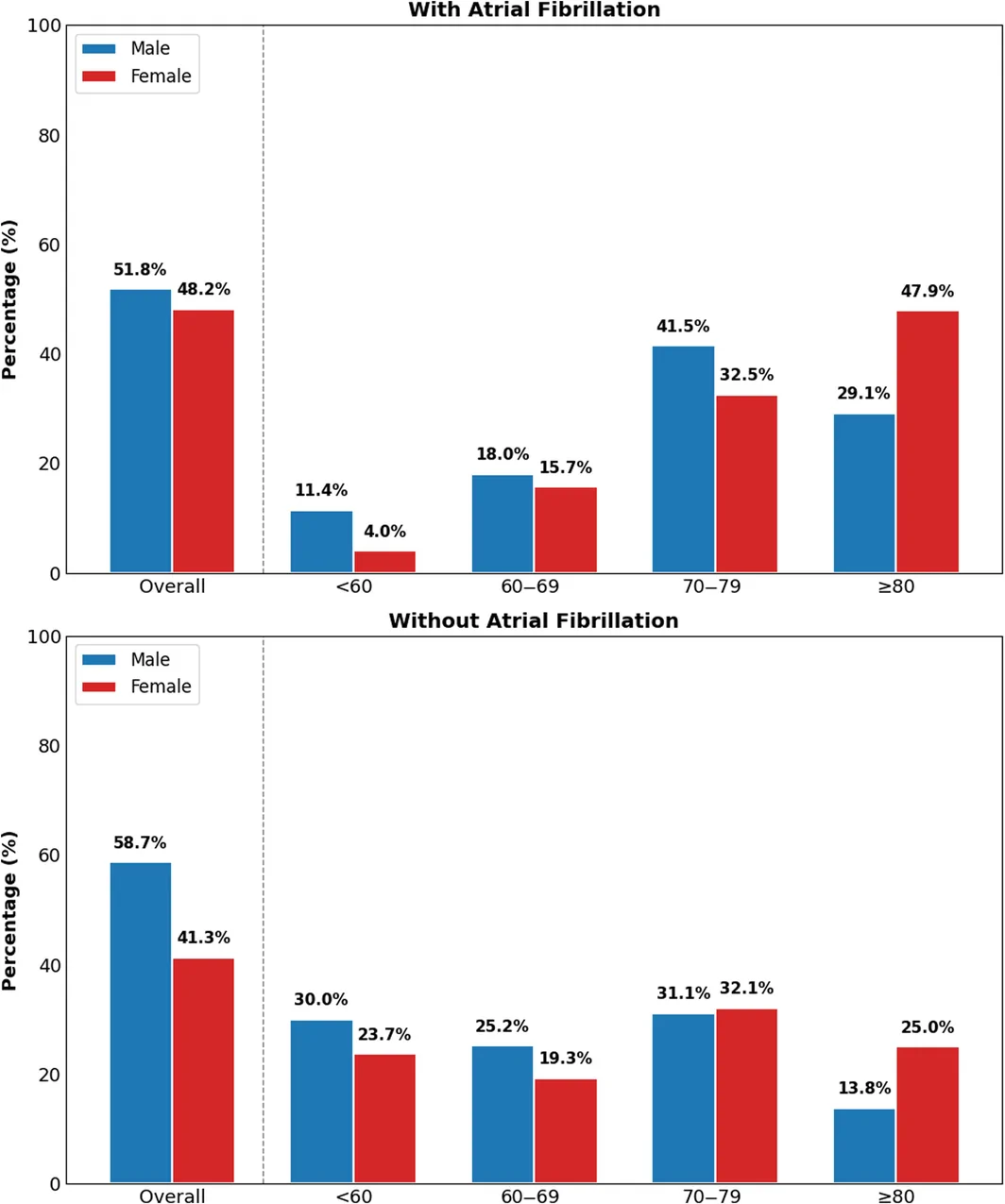
Distribution of sex and age for those populations with atrial fibrillation and without atrial fibrillation. sICH indicates symptomatic intracranial hemorrhage.

Women with AF had a significantly longer median (interquartile range) time from stroke onset to hospital arrival, to groin puncture, and to successful reperfusion/end of procedure, but similar time from arrival to successful reperfusion/end of procedure compared with men. For both sexes without AF, time metrics were similar.

#### Primary Outcome

Figure 3 and Table S3 shows the comparison of primary and secondary outcomes between patients with and without AF and between men and women.

**Figure 3:**
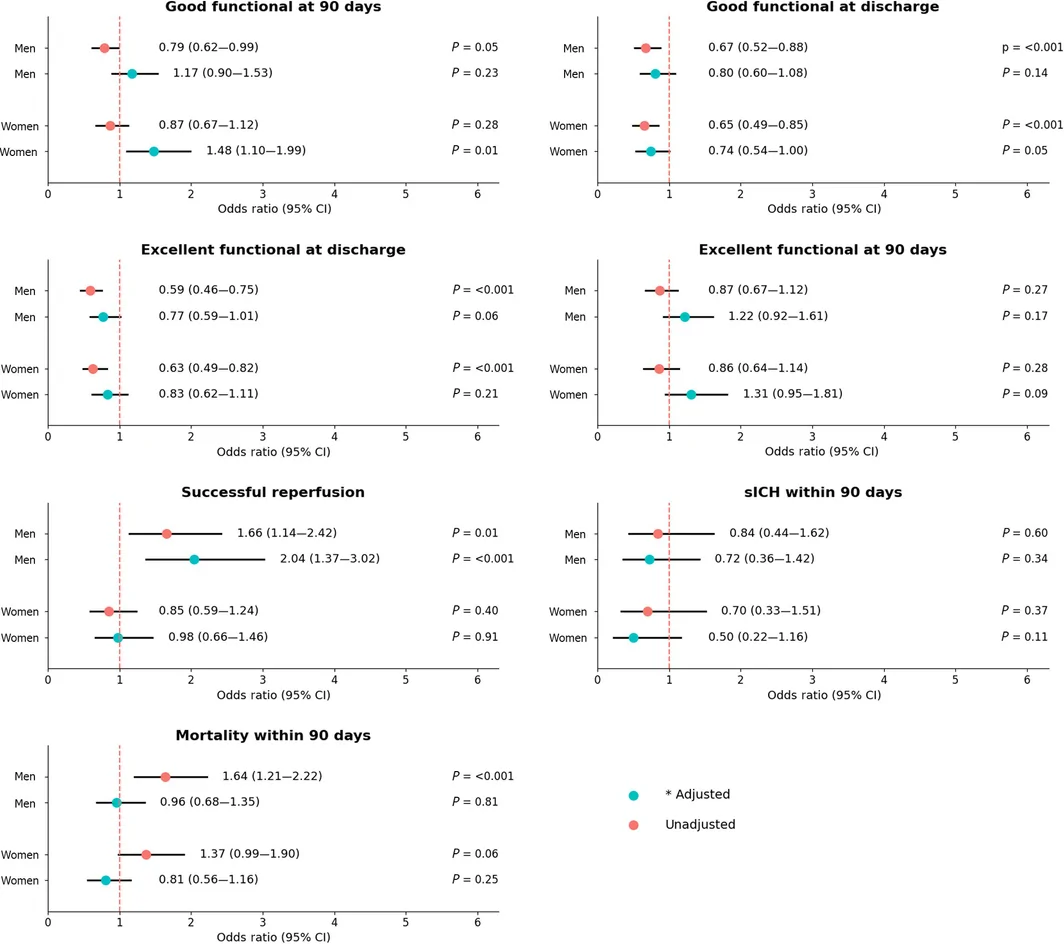
Outcomes comparison of the Danish Stroke Registry by atrial fibrillation status; stratified by sex. Excellent outcome and good outcome are defined as modified Rankin Scale score (0–1) and (0–2). SICH indicates symptomatic intracranial hemorrhage. *Adjusting for confounders included (age, cohabitation, immigration status, admission National Institutes of Health Stroke Scale score and Charlson Comorbidity Index [0, 1–2, or ≥3]); the reference is no atrial fibrillation compared with atrial fibrillation.

After adjusting for confounding factors, there was a statistically significant difference in good functional outcome at 90‐days in women with AF compared with women without AF (adjusted odds ratio [aOR], 1.48 [95% CI, 1.10–1.99]) but no significant difference in men with and without AF (aOR, 1.17 [95% CI, 0.90–1.53]).

#### Secondary Outcomes

After adjusting for confounding factors, men with AF had higher odds of successful reperfusion than men without AF (aOR, 2.04 [95% CI, 1.37–3.02]), whereas no association was observed in women (aOR, 0.98 [95% CI, 0.66–1.46]). AF was not associated with good functional outcome at discharge, excellent functional outcome at discharge or 90 days, sICH within 90 days, or 90‐day mortality in either sex (Figure 3).

### Subgroups Analysis

Among women, the proportion achieving a good functional outcome at 90 days was 65.9% in younger patients (age <72.5 years) with AF and 59.1% in younger patients without AF. Among older women (age ≥72.5 years), the corresponding proportions were 36.1% and 35.8%, respectively. Among men, the proportions achieving a good functional outcome at 90 days were comparable between patients with and without AF in both age groups.

Mortality within 90 days was numerically greater among older men and women compared with younger counterparts, while sICH rates were comparable across all age groups (Table 2; Figure 4). Among older men, successful reperfusion rates was observed in 89.9% of patients with AF and 76.9% of those without AF. No notable differences were observed among younger men and women with and without AF.

**Table 2:** Clinical Outcomes of Ischemic Stroke Treated With EVT by Atrial Fibrillation Status, Stratified by Sex and Median Age, Danish Stroke Registry

Outcome	Male sex				Female sex			
	Age, y <72.5 (n=821)		Age, y ≥72.5 (n=622)		Age, y <72.5 (n=450)		Age, y ≥72.5 (n=649)	
	AF	No AF	AF	No AF	AF	No AF	AF	No AF
No. (%)	143 (17.4)	678 (82.6)	235 (37.8)	387 (62.2)	88 (19.6)	362 (80.4)	263 (40.5)	386 (59.5)
Discharge mRS score, median (IQR)	0 (0–0)	0 (0–0)	0 (0–2)	0 (0–1)	0 (0–0)	0 (0–0)	0 (0–1)	0 (0–1)
90‐d mRS score, median (IQR)	2 (1–4)	2 (1–3.5)	3 (1–5)	3 (1–5)	2 (1–3)	2 (1–4)	3 (2–5)	3 (1–5)
Good functional status at 90‐d, n (%)	85 (59.4)	404 (59.6)	95 (40.4)	167 (43.2)	58 (65.9)	214 (59.1)	95 (36.1)	138 (35.8)
Good functional status at discharge, n (%)	101 (70.6)	541 (79.8)	169 (71.9)	298 (77.0)	65 (73.9)	275 (76.0)	164 (62.4)	281 (72.8)
Excellent functional status at discharge, n (%)	97 (67.8)	507 (74.8)	131 (55.7)	262 (67.7)	61 (69.3)	262 (72.4)	135 (51.3)	236 (61.1)
Excellent functional status at 90‐d, n (%)	58 (40.6)	255 (37.6)	58 (24.7)	105 (27.1)	35 (39.8)	131 (36.2)	59 (22.4)	93 (24.1)
Successful reperfusion (thrombolysis in cerebral infarction ≥2b), n (%)	120 (88.9)	549 (87.7)	205 (89.9)	279 (76.9)	75 (88.2)	292 (88.8)	210 (84)	304 (85.4)
Symptomatic intracranial hemorrhage within 90 d, n (%)	6 (4.2)	22 (3.2)	6 (2.6)	18 (4.7)	*	11 (3)	*	16 (4.1)
Mortality within 90 d, n (%)	19 (13.3)	60 (8.8)	59 (25.1)	86 (22.2)	9 (10.2)	35 (9.7)	61 (23.2)	80 (20.7)

Median age is 72.45.

AF indicates atrial fibrillation; EVT,endovascular thrombectomy; and IQR, interquartile range.

* Number of cases are not reported in accordance with Statistics Denmark's data confidentiality policy.

**Figure 4:**
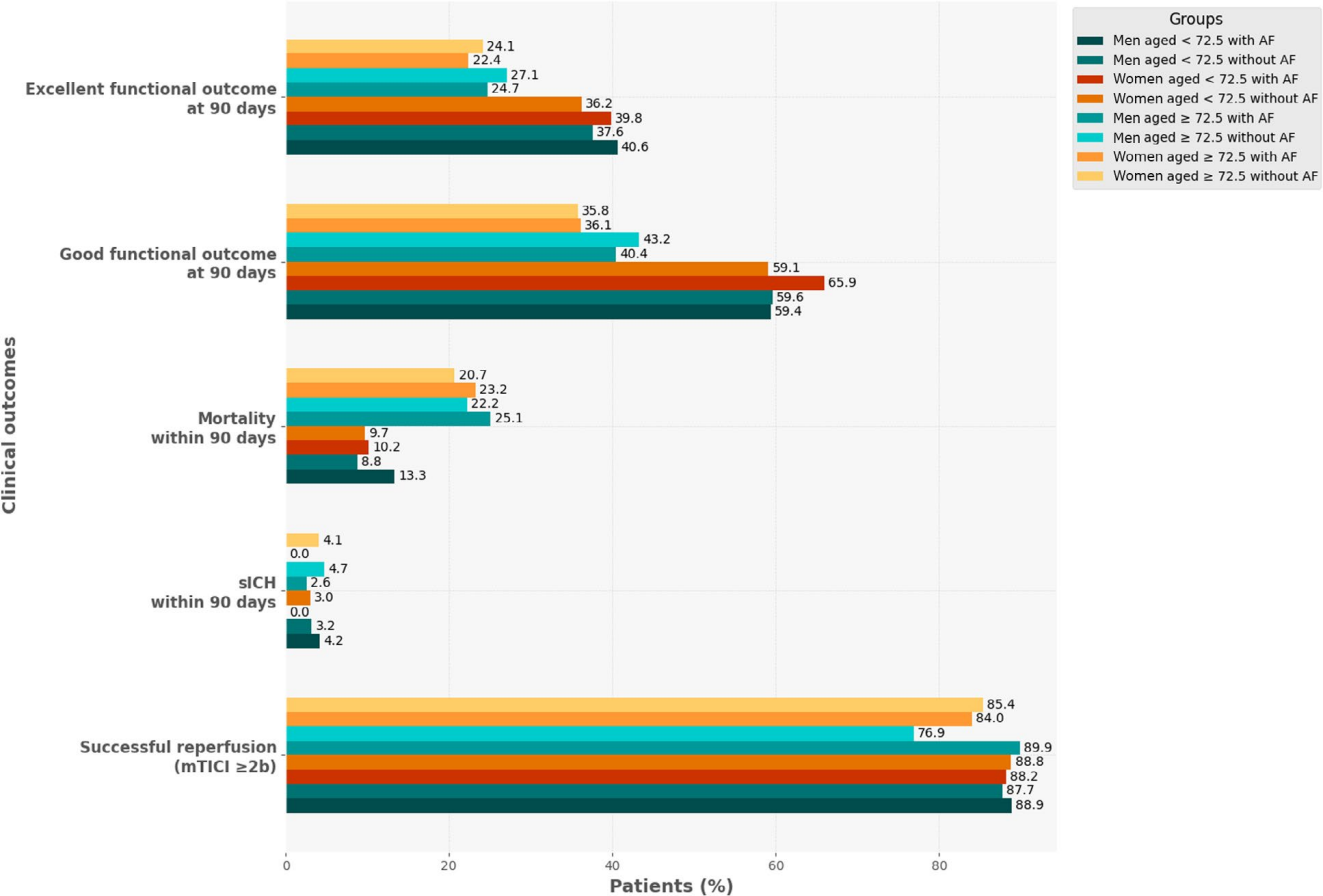
Clinical outcomes by atrial fibrillation status stratified by age and sex. Median age is 72.5. AF indicates atrial fibrillation; mTICI, modified thrombolysis in cerebral infarction scale; and SICH, symptomatic intracranial hemorrhage. *Number of cases are not reported in accordance with Statistics Denmark's data confidentiality policy.

### Effect Modification of Sex on Outcomes

Interaction terms (AF×sex) were included in the logistic regression models to assess for any effect modification of sex between groups with and without AF. There was a significant interaction effect of sex and successful reperfusion (*P*_interaction_<0.001) in older men with AF compared with older men without AF (aged ≥72.5 years) (aOR, 3.03 [95% CI, 1.82–5.04]) but not for women (aOR, [0.94 95% CI, 0.59–1.49]).

No interaction effect of sex was found for the remaining outcomes and age strata (all *P*_interaction_>0.05) (Table S4). No multicollinearity and no clinically significant effect modification were detected. Figure 5 shows a forest plot of the outcome ORs for AF stratified by sex and age over or under median age. Age strata were defined using the cohort median (72.5 years): <72.5 versus ≥72.5 years.

**Figure 5:**
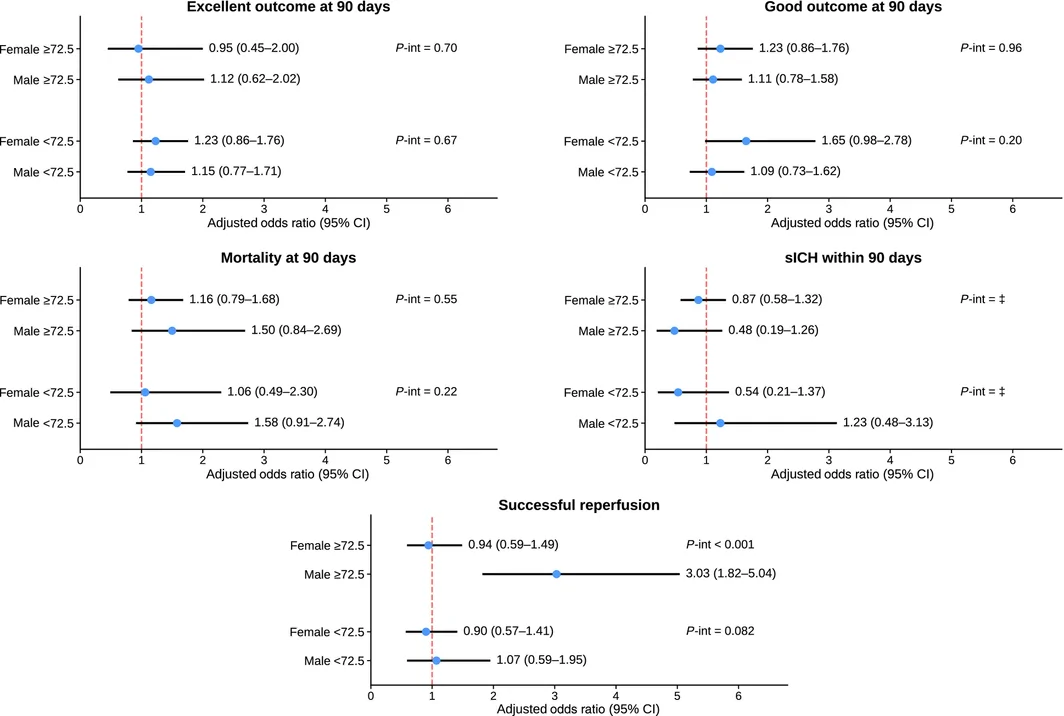
Interaction analyses of sex and median age in patients treated with EVT by atrial fibrillation status. Excellent outcome and good outcome are defined as modified Rankin Scale score (0–1) and (0–2). EVT indicates endovascular thrombectomy; SICH, symptomatic intracranial hemorrhage. *Adjusting for confounders included (age, cohabitation, immigration status, admission National Institutes of Health Stroke Scale score and Charlson Comorbidity Index [0, 1–2, or ≥3]); the reference is no atrial fibrillation compared with atrial fibrillation. ^‡^Number of cases are not reported in accordance with Statistics Denmark's data confidentiality policy.

### Sensitivity Analysis

In the fully adjusted logistic regression model, women with AF were associated with higher odds of achieving a good functional outcome compared with women without AF (aOR, 1.48 [95% CI, 1.10–1.99]). However, in the unadjusted analysis, there was no significant difference in the odds of good functional outcome between women with and without AF (unadjusted OR, 0.87 [95% CI, 0.67–1.12]). Given this apparent reversal in the direction of the association after covariate adjustment, additional sensitivity analyses were performed to better understand the influence of individual covariates, particularly age. When adjusting for age alone, the OR increased to 1.27 (95% CI, 0.97–1.68), indicating that age adjustment alone explained a substantial portion of the shift in the association. In contrast, when adjusting for all prespecified covariates except age, the association between AF and good functional outcome was attenuated, with an OR of 1.10 (95% CI, 0.84–1.46). Further analyses examining adjustment for individual covariates, such as NIHSS score, cohabitation status, immigration status, or CCI produced only modest changes in the OR, which remained close to the unadjusted estimate (point estimate ∼0.9 in most cases).

## DISCUSSION

This nationwide study from the Danish Stroke Registry found that women with AF had higher adjusted odds of good functional outcome at 90 days compared with women without AF. In contrast, men had similar rates of good functional outcomes at 90 days regardless of AF status. There was no statistically significant difference in sICH or mortality between men with and without AF compared with women with and without AF. Second, men with AF had significantly higher rates of successful reperfusion compared with men without AF, which was not observed in women. Third, older men with AF were more likely to have successful reperfusion than older men without AF, while no similar benefit was observed in older women.

These findings, based on unselected patients residing in Denmark who underwent thrombectomy, align with a previous study investigating the impact of sex on EVT outcomes, showing a trend toward better adjusted good functional outcome at 90 days in women compared with men, whereas sICH and mortality were similar in both sexes.^24^ In contrast to prior clinical trial cohorts that enrolled highly selected patients with fewer comorbidities, our real‐world data reveal nuanced age‐ and sex‐specific patterns that may be obscured in controlled trial settings. Our results further suggest the importance of age‐ and sex‐specific variation in modulating the effect of AF on EVT outcomes.

Sex‐related disparities in the baseline characteristics, such as age, comorbidities, education, and living status, may partly explain sex differences in underlying pathophysiology and outcomes. In our cohort, women with AF were older, more often had hypertension, and experienced longer prehospital delays compared with men. These factors may explain why women with AF had a lower point estimate of the OR for good functional outcomes at 90 days than women without AF before adjustment. A previous study has shown that women are more often admitted late, partly because they more often lived alone and are discovered later after stroke onset.^13^

In our study, the unadjusted model suggested no significant difference in good functional outcomes at 90‐days in women with and without AF. However, after adjusting for key covariates, particularly age, the direction of the association reversed, with women with AF showing significantly higher odds of good functional outcome at 90 days than women without AF. Age acted as a strong confounder, as women with AF were significantly older in our cohort. Age‐adjusted models alone (OR, 1.27) accounted for most of the shift, while adjusting for other variables produced only modest changes. This illustrates the critical role of age in outcome modeling and how residual confounding may persist despite adjustment in observational data.

Younger women with AF had a higher rate of good functional outcomes than those without AF (65.9% versus 59.1%). This finding may reflect differences in stroke mechanism (cardioembolic versus noncardioembolic), thrombus composition, and treatment workflow. Although timely anticoagulation could potentially contribute to improved outcomes, we did not evaluate anticoagulation use by age and sex in this cohort; this hypothesis warrants further investigation. In contrast, older women showed no such advantage of good functional outcomes (36.1% versus 35.8%).

Notably, workflow time metrics were consistently longer among patients without AF, including onset‐to‐groin puncture and arrival‐to‐reperfusion times. Treatment delays are strongly associated with infarct progression and functional recovery after EVT and may therefore partly contribute to outcome differences between patients with and without AF. However, these timing variables may also reflect differences in stroke mechanism and care pathways and may lie on the causal pathway rather than acting solely as confounders. As such, they were not included in the primary multivariable models, and residual confounding related to workflow delays cannot be excluded.

The higher rate of successful reperfusion in men with AF compared with men without AF may have several potential explanations. Difficult vessel access and tortuous arteries are often a product of long‐standing hypertension, older age, and vascular comorbidities. Younger men with AF might have easier access and straighter arteries, providing better conditions for the neurointerventionalist. Differences in thrombus characteristics and the embolic nature of AF‐related strokes could also play a role. In addition, AF‐related strokes are more often cardioembolic, whereas patients without AF may have a higher proportion of atherosclerotic or mixed‐mechanism occlusions, which could be associated with more complex procedures and lower reperfusion success. This pattern is not observed in women, possibly due to age and biological differences, hormonal factors, comorbidity burden, and varying responses to AF and EVT. Residual confounding may also contribute, particularly from unmeasured procedural and anatomical factors such as clot burden, collateral status, and number of thrombectomy passes. Identifying modifiable procedural and clinical factors contributing to reperfusion success may help optimize EVT strategies across age‐ and sex‐specific risk groups.

Although older men with AF had higher rates of successful reperfusion, this did not translate into higher odds of good functional outcome. Angiographic reperfusion is necessary but may be insufficient for functional recovery, particularly in older patients in whom outcomes are strongly influenced by baseline frailty, comorbidity burden, infarct size, collateral status, periprocedural complications, and poststroke care and rehabilitation. These factors may attenuate the functional impact of reperfusion differences across groups.

Our findings add nuance to studies like the pooled analyses^15^ from SWIFT (Solitaire With the Intention for Thrombectomy), STAR (Solitaire Flow Restoration Thrombectomy for Acute Revascularization), and SWIFT PRIME (Solitaire With the Intention for Thrombectomy as Primary Endovascular Treatment) cohorts, which reported that both sexes had comparable rates of good functional outcomes, but women were consistently older and had a higher prevalence of AF at baseline.^15^ In contrast to these highly selected randomized clinical trial populations with limited comorbidity burden, our population‐based data suggest that AF interacts with age and sex to influence EVT outcomes, relationships that may not be apparent in trial cohorts. Our study further suggests that only women with AF had better functional outcomes (mRS score ≤2) at 90 days after adjustment compared with women without AF, whereas men with AF were more often successfully reperfused compared with men without AF, especially with advancing age. Among men, the absence of functional outcome differences by AF status may reflect a higher prevalence of competing stroke mechanisms or mixed causes (eg, large‐artery atherosclerosis) and a greater vascular risk burden, potentially diminishing any differential effect attributable to AF alone.

Although AF is typically associated with worse outcomes due to its association with more severe strokes, our adjusted analyses showed that women with AF had higher odds of good functional outcome than women without AF. This counterintuitive finding emphasizes the complexity of stroke pathophysiology and suggests that unmeasured factors, such as prestroke disability status, access to care, or differences in rehabilitation, could play a role.

These findings highlight the importance of identifying modifiable clinical and sociodemographic factors to guide personalized treatment and postacute care and demonstrate the need to consider sex and age interactions when developing secondary prevention strategies and optimizing EVT pathways.

In summary, AF was associated with better adjusted functional outcomes among women but not men, whereas successful reperfusion was higher among men with AF, particularly at older ages. These findings highlight potentially important age‐ and sex‐specific patterns that warrant confirmation in other cohorts incorporating more granular procedural and pathogenetic data.

### Strengths and Limitations

This study has several strengths including its large, nationwide, real‐world cohort with high‐quality, prospectively collected registry data and minimal missingness. The high sensitivity of stroke diagnosis recorded in the Danish Stroke Registry (91%–97%) adds to the reliability of the study.^20^, ^21^ Moreover, the study included a broad range of clinical and sociodemographic factors with few missing data, contributing valuable knowledge about sex disparities in EVT outcomes among patients with AF compared with patients without AF.

However, there are some limitations. The observational design limits causal inference, and there remains potential for residual confounding from unmeasured variables, such as prestroke disability, collateral status, procedural parameters, and psychological or social factors. In addition, the registry did not capture procedural granularity such as the number of thrombectomy passes/attempts, limiting assessment of technical complexity as a contributor to reperfusion differences. Furthermore, workflow time metrics differed between groups with and without AF but were not included in the primary adjusted models, and residual confounding related to treatment delays may therefore remain. We also lacked data on biological factors such as sex hormone status and other measures of physiological reserve that may influence recovery trajectories.^25^ Although anticoagulation is an important component of AF management, we did not observe significant sex‐based differences in anticoagulation therapy in our cohort, and anticoagulation use stratified by age was not specifically assessed. Cause‐specific mortality was not available; therefore, we assessed all‐cause mortality at 90 days. The Danish Stroke Registry, established as a quality improvement initiative, has missing data values due to data collection being integrated as part of routine clinical work. However, a structured audit process is executed annually to assess and maintain the quality of the database.^19^ Future studies incorporating detailed measures of AF management, prestroke function, and poststroke rehabilitation could clarify these observed interactions and guide sex‐ and age‐specific treatment pathways.

## CONCLUSIONS

In this nationwide study of patients undergoing EVT for large vessel occlusion strokes in Denmark, women with AF had higher adjusted odds of a good functional outcome at 90 days compared with women without AF, which was not observed in men. No significant sex differences in clinical outcomes were found between groups with and without AF. Age‐stratified analyses indicated that men with AF were most likely to achieve successful reperfusion with advancing age. These findings suggest the presence of possible age‐ and sex‐specific patterns in EVT outcomes associated with AF, which may indicate untapped potential to improve functional outcomes, particularly among men with AF. However, given the observational design and potential for residual confounding, these results should be interpreted as exploratory and hypothesis generating rather than definitive evidence of causality. Further studies are needed to clarify the biological, clinical, and procedural mechanisms that may contribute to these patterns, and to determine whether targeted interventions could optimize outcomes across different demographic subgroups.

## Sources of Funding

Muath Alobaida was funded by a PhD studentship from King Saud University.

## Disclosures

Deirdre A. Lane received investigator‐initiated educational grants from Bristol‐Myers Squibb and Pfizer, outside the submitted work. She is a co‐applicant on the AFFIRMO project on multimorbidity in AF (Grant Agreement No. 899871), ARISTOTELES project on artificial intelligence for management of chronic long‐term conditions (Grant Agreement No. 101080189), and the TARGET project on digital twins for personalized management of atrial fibrillation and stroke (Grant Agreement No. 101136244), all of which are funded by the European Union Horizon Europe Research & Innovation program. Gregory Y. H. Lip is a consultant and speaker for BMS/Pfizer, Boehringer Ingelheim, Daiichi‐Sankyo, Anthos. No fees are received personally. He is a National Institute for Health and Care Research Senior Investigator and co‐principal investigator of the AFFIRMO project on multimorbidity in AF (grant agreement No 899871), TARGET project on digital twins for personalized management of atrial fibrillation and stroke (grant agreement No 101136244) and ARISTOTELES project on artificial intelligence for management of chronic long term conditions (grant agreement No 101080189), which are all funded by the European Union's Horizon Europe Research & Innovation program. Thomas Truelsen Speaker honoraria from Pfizer/Bristol Myers Squibb/Boehringer‐Ingelheim/Astra Zeneca outside the submitted work and participated in research outside the submitted work funded by Novo Nordisk. David Gaist received speaker honoraria from Pfizer and Bristol Myers Squibb outside the submitted work and participated in research outside the submitted work funded by Bayer with funds paid to the institution where he is employed. Stephanie L. Harrison has received grant funding from Bristol‐Myers Squibb outside of the submitted work. Søren P. Johnsen is co‐principal investigator of the AFFIRMO project on multimorbidity in AF (European Union Horizon grant agreement No 899871) and has provided consultant work for Bristol‐Myers Squibb. The remaining authors have no disclosures to report.

## Supporting information

Tables S1–S4

STROBE Statement
