# Supplementary material for: Sex and Age Differences in Outcomes After Endovascular Thrombectomy of Patients With Acute Ischemic Stroke and Atrial Fibrillation: A Danish Nationwide Study
Source: J Am Heart Assoc. 2026 Aug 7;15(16):e048384. doi: 10.1161/JAHA.125.048384 (PMC13695746; doi:10.1161/JAHA.125.048384)
Supplement: Supplementary file 2 [file JAH3-15-e048384-s002.pdf]

Supplemental Material

Table S1. Baseline characteristics of the Danish Stroke Registry by atrial fibrillation status, stratified by sex.

	**Atrial fibrillation**				**No atrial fibrillation**			
**Characteristic**	**Total (n=729)**	**Male (n=378)**	**Female (n=351)**	***p***	**Total (n=1,813)**	**Male (n=1,065)**	**Female (n=748)**	***p***
Demographic factors								
**Age group (years), n (%)**								
<60	57	43 (11.4)	14 (4.0)	<0.001	496	319 (30.0)	177 (23.7)	<0.001
60-69	123	68 (18.0)	55 (15.7)		412	268 (25.2)	144 (19.3)	
70-79	271	157 (41.5)	114 (32.5)		571	331 (31.1)	240 (32.1)	
≥80	278	110 (29.1)	168 (47.9)		334	147 (13.8)	187 (25.0)	
**Education, n (%)***								
Low	302	132 (34.9)	170 (48.4)	<0.001	644	306 (28.7)	338 (45.2)	<0.001
Medium	261	156 (41.3)	105 (29.9)		762	500 (46.9)	262 (35.0)	
High	150	82 (21.7)	68 (19.4)		371	239 (22.4)	132 (17.6)	
**Type of residence, n (%)**								
Own residence	696	366 (96.8)	330 (94.0)	***	1725	1,016 (95.4)	709 (94.8)	0.086
Care home	***	***	12 (3.4)		28	11 (1.0)	17 (2.3)	
Undisclosed	18	9 (2.4)	9 (2.6)		60	38 (3.6)	22 (2.9)	
**Marital status, n (%)**								
Cohabitant	438	274 (72.5)	164 (46.7)	<0.001	1239	798 (74.9)	441 (59.0)	<0.001
Lives alone	275	96 (25.4)	179 (51.0)		506	228 (21.4)	278 (37.2)	
Undisclosed	16	8 (2.1)	8 (2.3)		68	39 (3.7)	29 (3.9)	
Lifestyle prognostic factors								
**Smoking, n (%)**								
Current or Ex-smoker	317	201 (53.2)	116 (33.0)	<0.001	871	537 (50.4)	334 (44.7)	0.031
Never	240	102 (27.0)	138 (39.3)		551	308 (28.9)	243 (32.5)	
Undisclosed	172	75 (19.8)	97 (27.6)		391	220 (20.7)	171 (22.9)	
**Alcohol, items/week †**				0.007				<0.001
7-14 items-week or fewer	504	264 (69.8)	240 (68.4)		1273	725 (68.1)	548 (73.3)	
Over 7-14 items-week	56	40 (10.6)	16 (4.6)		164	121 (11.4)	43 (5.7)	

Clinical prognostic factors								
Admission NIHSS score, median (IQR)‡	708	16 (11–21)	17 (12–22)	0.15	1,733	15 (8–19)	16 (10–20)	0.057
**Charlson Comorbidity index, n (%)**								
Low (0 points)	352	171 (45.2)	181 (51.6)	0.17	1146	665 (62.4)	481 (64.3)	0.68
Moderate (1-2 points)	249	133 (35.2)	116 (33.0)		477	284 (26.7)	193 (25.8)	
High (≥3 points)	128	74 (19.6)	54 (15.4)		190	116 (10.9)	74 (9.9)	
Transient ischemic attack, n (%)	18	10 (2.6)	8 (2.3)	0.75	***	9 (0.8)	***	***
Hypertension, n (%)§	418	200 (53.6)	218 (62.8)	0.012	868	480 (45.6)	388 (52.9)	0.003
Diabetes, n (%)‖	97	58 (15.5)	39 (11.1)	0.082	213	139 (13.2)	74 (10.1)	0.050
Myocardial infarction, n (%)#	81	51 (13.7)	30 (8.6)	0.033	124	95 (9.0)	29 (4.0)	<0.001
Peripheral vascular disease, n (%)**	37	22 (6.0)	15 (4.4)	0.35	73	51 (4.9)	22 (3.0)	0.048
**Occlusion site, n (%)**								
Common carotid artery	***	***	***	***	19	10 (0.9)	9 (1.2)	0.59
Internal carotid artery	74	42 (11.1)	32 (9.1)	0.37	414	269 (25.3)	145 (19.4)	0.003
Internal carotid artery-tandem	80	38 (10.1)	42 (12.0)	0.41	186	99 (9.3)	87 (11.6)	0.11
Middle cerebral artery- M1	376	183 (48.4)	193 (55.0)	0.076	829	455 (42.7)	374 (50.0)	0.002
Middle cerebral artery- M2	174	91 (24.1)	83 (23.6)	0.89	382	227 (21.3)	155 (20.7)	0.76
Anterior cerebral artery- A1 or A2	20	12 (3.2)	8 (2.3)	0.46	43	28 (2.6)	15 (2.0)	0.39
Vertebral artery	***	7 (1.9)	***	***	43	34 (3.2)	9 (1.2)	0.006
Basilar artery	37	24 (6.3)	13 (3.7)	0.10	151	106 (10.0)	45 (6.0)	0.003
Posterior cerebral artery /other	38	23 (6.1)	15 (4.3)	0.27	86	56 (5.3)	30 (4.0)	0.22
Process of care								
IV Thrombolysis, n (%)	343	175 (46.3)	168 (47.9)	0.67	1108	660 (62.0)	448 (59.9)	0.37
Anticoagulation therapy, n (%)	271	149 (39.4)	122 (34.8)	0.19	63	39 (3.7)	24 (3.2%)	0.60
**Type of anesthesia, n (%)††**								
General anesthesia	522	277 (73.3)	245 (69.8)	0.20	1329	777 (73.0)	552 (73.8)	0.53
Conscious anesthesia	191	91 (24.1)	100 (28.5)		417	251 (23.6)	166 (22.2)	
Time metrics, hours, median IQR								
Onset to hospital arrival ‡‡		3.2 (2.2–5.1)	3.5 (2.3–6.2)	0.007		3.6 (2.3–6.1)	3.8 (2.5–6.8)	0.15
Onset to groin puncture §§		3.5 (2.5–5.4)	3.9 (2.7–6.6)	0.006		4.0 (2.7–6.3)	4.2 (2.9–7.2)	0.080

Onset to successful reperfusion/end of procedure ‖‖	4.4 (3.3–6.6)	4.9 (3.6–7.6)	0.008	5.2 (3.8–7.7)	5.3 (3.9–8.2)	0.37
Arrival to successful reperfusion/end of procedure ##	2.8 (2.1–3.7)	2.8 (2.1–3.8)	0.87	3.2 (2.3–4.4)	3.2 (2.2–4.4)	0.64

IQR, interquartile range; IV, intravenous; mRS, modified Rankin Scale; NIHSS, National Institutes of Health Stroke Scale. Education was defined according to the International Standard Classification of Education. Redemption of anticoagulation drugs within 6 months before stroke. ***Number of cases are not reported in accordance with Statistics Denmark's data confidentiality policy. * 52 missing , † 545 missing , ‡ 102 missing, § 36 missing, ‖ 31 missing, # 38 missing, ** 75 missing, †† 83 missing, ‡‡ 128 missing, §§ 120 missing, ‖‖ 120 missing, ## 124 missing.

Table S2. Baseline characteristics of the Danish Stroke registry stratified by atrial fibrillation status.

**Characteristic**	**Atrial fibrillation (n=729)**	**No atrial fibrillation (n=1,813)**	***p***
Demographic factors			
Male, n (%)	378 (51.8)	1,065 (58.7)	0.001
**Age group, n (%)**			
**<60 y**	57 (7.8)	496 (27.4)	<0.001
**60-69 y**	123 (16.9)	412 (22.7)	
**70-79 y**	271 (37.2)	571 (31.5)	
≥80 y	278 (38.1)	334 (18.4)	
**Education, n (%)***			
Low	302 (41.4)	644 (35.5)	0.007
Medium	261 (35.8)	762 (42.0)	
High	150 (20.6)	371 (20.5)	
**Type of residence, n (%)**			
Own residence	696 (95.5)	1,725 (95.1)	***
Care home	***	28 (1.5)	
Undisclosed	18 (2.5)	60 (3.3)	
**Marital status, n (%)**			
Cohabitant	438 (60.1)	1,239 (68.3)	<0.001
Lives alone	275 (37.7)	506 (27.9)	
Undisclosed	16 (2.2)	68 (3.8)	
Lifestyle prognostic factors			
**Smoking, n (%)**			
Current or ex-smoker	317 (43.5)	871 (48.0)	0.076
Never	240 (32.9)	551 (30.4)	
Undisclosed	172 (23.6)	391 (21.6)	
**Alcohol, items/week †**			
7-14 items-week or fewer	504 (69.1)	1,273 (70.2)	0.37
Over 7-14 items-week	56 (7.7)	164 (9.0)	

Clinical prognostic factors			
Admission NIHSS score , median (IQR) ‡	17 (12–22)	15 (9–20)	<0.001
**Charlson Comorbidity index, n (%)**			
Low (0 points)	352 (48.3)	1,146 (63.2)	<0.001
Moderate (1-2 points)	249 (34.2)	477 (26.3)	
High (≥3 points)	128 (17.6)	190 (10.5)	
Transient ischemic attack, n (%)	18 (2.5)	***	***
Hypertension, n (%)§	418 (58.1)	868 (48.6)	<0.001
Diabetes, n (%)‖	97 (13.4)	213 (11.9)	0.30
Myocardial infarction, n (%)#	81 (11.3)	124 (7.0)	<0.001
Peripheral vascular disease, n (%)**	37 (5.3)	73 (4.1)	0.23
**Occlusion site, n (%)**			
Common carotid artery	***	19 (1.0)	***
Internal carotid artery	74 (10.2)	414 (22.8)	<0.001
Internal carotid artery-tandem	80 (11.0)	186 (10.3)	0.59
Middle cerebral artery- M1	376 (51.6)	829 (45.7)	0.008
Middle cerebral artery- M2	174 (23.9)	382 (21.1)	0.12
Anterior cerebral artery- A1 or A2	20 (2.7)	43 (2.4)	0.59
Vertebral artery	***	43 (2.4)	***
Basilar artery	37 (5.1)	151 (8.3)	0.005
Posterior cerebral artery /other	38 (5.2)	86 (4.7)	0.62
Process of care			
IV Thrombolysis, n (%)	343 (47.1)	1,108 (61.1)	<0.001
Anticoagulation therapy, n (%)	271 (37.2)	63 (3.5)	<0.001
**Type of anesthesia, n (%)††**			
General anesthesia	522 (71.6)	1,329 (73.3)	0.13
Conscious anesthesia	191 (26.2)	417 (23.0)	
Time metrics, hour, median IQR			
Onset to arrival at EVT hospital ‡‡	3.3 (2.3–5.5)	3.7 (2.4–6.4)	0.003
Onset to groin puncture §§	3.6 (2.6–5.9)	4.1 (2.8–6.7)	0.001
Onset to successful reperfusion/end of procedure ‖‖	4.6 (3.5–6.9)	5.3 (3.8–7.9)	<0.001

Arrival to successful reperfusion/end of procedure ##	2.8 (2.1–3.8)	3.2 (2.2–4.4)	<0.001

IQR, interquartile range; IV, intravenous; mRS, modified Rankin Scale; NIHSS, National Institutes of Health Stroke Scale. Education was defined according to the International Standard Classification of Education. Redemption of anticoagulation drugs within 6 months before stroke. ***Number of cases are not reported in accordance with Statistics Denmark's data confidentiality policy. * 52 missing , † 545 missing , ‡ 102 missing, § 36 missing, ‖ 31 missing, # 38 missing, ** 75 missing, †† 83 missing, ‡‡ 128 missing, §§ 120 missing, ‖‖ 120 missing, ## 124 missing.

Table S3. Outcomes comparison of the Danish Stroke Registry by atrial fibrillation status, stratified by sex.

Outcomes	Male (n=1,443)						Female (n=1,099)					
	AF (n=378)	No AF (n=1,065)	Unadjusted OR (95% CI)†	*p*	Adjusted* OR (95% CI)†	*p*	AF (n=351)	No AF (n=748)	Unadjusted OR (95% CI)†	*p*	Adjusted* OR (95% CI)†	*p*
Good functional status at 90-day, n (%)	180 (47.6)	571 (53.6)	0.79 (0.62–0.99)	0.05	1.17 (0.90–1.53)	0.23	153 (43.6)	352 (47.1)	0.87 (0.67–1.12)	0.28	1.48 (1.10–1.99)	0.01
Good functional status at discharge, n (%)	270 (71.4)	839 (78.8)	0.67 (0.52–0.88)	<0.001	0.80 (0.60–1.08)	0.14	229 (65.2)	556 (74.3)	0.65 (0.49–0.85)	<0.001	0.74 (0.54–1.00)	0.05
Excellent functional status at discharge, n (%)	228 (60.3)	769 (72.2)	0.59 (0.46–0.75)	<0.001	0.77 (0.59–1.01)	0.06	196 (55.8)	498 (66.6)	0.63 (0.49–0.82)	<0.001	0.83 (0.62–1.11)	0.21
Excellent functional status at 90-day, n (%)	116 (30.7)	360 (33.8)	0.87 (0.67–1.12)	0.27	1.22 (0.92–1.61)	0.17	94 (26.8)	224 (29.9)	0.86 (0.64–1.14)	0.28	1.31 (0.95–1.81)	0.09
Successful reperfusion (TICI ≥2b), n (%)	325 (86.0)	828 (77.7)	1.66 (1.14–2.42)	0.01	2.04 (1.37–3.02)	<0.001	285 (81.2)	596 (79.7)	0.85 (0.59–1.24)	0.40	0.98 (0.66–1.46)	0.91
sICH within 90 days, n (%)	12 (3.2)	40 (3.8)	0.84 (0.44–1.62)	0.60	0.72 (0.36–1.42)	0.34	9 (2.6)	27 (3.6)	0.70 (0.33–1.51)	0.37	0.50 (0.22–1.16)	0.11
Mortality within 90 days, n (%)	78 (20.6)	146 (13.7)	1.64 (1.21–2.22)	<0.001	0.96 (0.68–1.35)	0.81	70 (19.9)	115 (15.4)	1.37 (0.99–1.90)	0.06	0.81 (0.56–1.16)	0.25

AF, Atrial fibrillation; IQR, interquartile range; mRS, modified Rankin Scale; OR, odds ratio; SICH, symptomatic intracranial hemorrhage; TICI, thrombolysis in cerebral infarction scale. *Adjusting for confounders included (age, cohabitation, immigration status, admission National Institutes of Health Stroke Scale and whether Charlson Comorbidity Index [0, 1-2, or ≥ 3]. OR for atrial fibrillation. † The reference is no AF.

Table S4. Interaction analyses of sex stratified outcomes in patients treated with EVT by atrial fibrillation status, Danish Stroke Registry.

Outcome	Age	Male OR (95% CI)†	Female OR (95% CI)†	*P interaction*
90-day good outcome	< 72.5	Crude: 0.99 (0.69–1.44)	Crude: 1.34 (0.82–2.18)	0.34
		Adj*: 1.09 (0.73–1.62)	Adj*: 1.65 (0.98–2.78)	0.20
	≥ 72.5	Crude: 0.89 (0.64–1.24)	Crude: 1.02 (0.73–1.41)	0.59
		Adj*: 1.11 (0.78–1.58)	Adj*: 1.23 (0.86–1.76)	0.96
90-day excellent outcome	< 72.5	Crude: 1.13 (0.78–1.64)	Crude: 1.16 (0.72–1.88)	0.93
		Adj*: 1.26 (0.85–1.88)	Adj*: 1.42 (0.86–2.34)	0.67
	≥ 72.5	Crude: 0.88 (0.61–1.28)	Crude: 0.91 (0.63–1.32)	0.90
		Adj*: 1.15 (0.77–1.71)	Adj*: 1.12 (0.74–1.68)	0.70
Successful reperfusion	< 72.5	Crude: 1.12 (0.62–2.02)	Crude: 0.95 (0.45–2.00)	0.67
		Adj*: 1.07 (0.59–1.95)	Adj*: 1.01 (0.46–2.23)	0.82
	≥ 72.5	Crude: 2.68 (1.64–4.40)	Crude: 0.90 (0.57–1.41)	<0.001
		Adj*: 3.03 (1.82–5.04)	Adj*: 0.94 (0.59–1.49)	<0.001
sICH within 90 days	< 72.5	Crude: 1.31 (0.52–3.28)	Crude: ‡	‡
		Adj*: 1.23 (0.48–3.13)	Adj: ‡	‡
	≥ 72.5	Crude: 0.54 (0.21–1.37)	Crude: ‡	‡
		Adj*: 0.48 (0.19–1.26)	Adj: ‡	‡
Mortality within 90 days	< 72.5	Crude: 1.58 (0.91–2.74)	Crude: 1.06 (0.49–2.30)	0.42
		Adj*: 1.50 (0.84–2.69)	Adj*: 0.75 (0.32–1.74)	0.22
	≥ 72.5	Crude: 1.17 (0.80–1.72)	Crude: 1.16 (0.79–1.68)	0.95
		Adj*: 0.87 (0.58–1.32)	Adj*: 1.03 (0.69–1.54)	0.55

Median age is 72.5. mRS, modified Rankin Scale; OR, odds ratio; SICH, symptomatic intracranial hemorrhage. *Adjusting for confounders included (age, cohabitation, immigration status, admission National Institutes of Health Stroke Scale and whether Charlson Comorbidity Index [0, 1-2, or ≥ 3]. † The reference is no AF and OR is for atrial fibrillation. ‡ Number of cases are not reported in accordance with Statistics Denmark's data confidentiality policy.
